# Short-Course, High-Dose Rifampicin Achieves *Wolbachia* Depletion Predictive of Curative Outcomes in Preclinical Models of Lymphatic Filariasis and Onchocerciasis

**DOI:** 10.1038/s41598-017-00322-5

**Published:** 2017-03-16

**Authors:** Ghaith Aljayyoussi, Hayley E. Tyrer, Louise Ford, Hanna Sjoberg, Nicolas Pionnier, David Waterhouse, Jill Davies, Joanne Gamble, Haelly Metuge, Darren A. N. Cook, Andrew Steven, Raman Sharma, Ana F. Guimaraes, Rachel H. Clare, Andrew Cassidy, Kelly L. Johnston, Laura Myhill, Laura Hayward, Samuel Wanji, Joseph D. Turner, Mark J. Taylor, Stephen A. Ward

**Affiliations:** 1 Department of Parasitology, Liverpool School of Tropical Medicine, Pembroke Place, Liverpool L3 5QA UK; 2 Research Foundation in Tropical Medicine and the Environment, Buea, Cameroon; 3 Department of Microbiology and Parasitology, University of Buea, Buea, Cameroon

## Abstract

Lymphatic filariasis (LF) and onchocerciasis are priority neglected tropical diseases targeted for elimination. The only safe drug treatment with substantial curative activity against the filarial nematodes responsible for LF (*Brugia malayi, Wuchereria bancrofti*) or onchocerciasis (*Onchocerca volvulus*) is doxycycline. The target of doxycycline is the essential endosymbiont, *Wolbachia.* Four to six weeks doxycycline therapy achieves >90% depletion of *Wolbachia* in worm tissues leading to blockade of embryogenesis, adult sterility and premature death 18–24 months post-treatment. Long treatment length and contraindications in children and pregnancy are obstacles to implementing doxycycline as a public health strategy. Here we determine, via preclinical infection models of *Brugia malayi* or *Onchocerca ochengi* that elevated exposures of orally-administered rifampicin can lead to *Wolbachia* depletions from filariae more rapidly than those achieved by doxycycline. Dose escalation of rifampicin achieves >90% *Wolbachia* depletion in time periods of 7 days in *B. malayi* and 14 days in *O. ochengi*. Using pharmacokinetic-pharmacodynamic modelling and mouse-human bridging analysis, we conclude that clinically relevant dose elevations of rifampicin, which have recently been determined as safe in humans, could be administered as short courses to filariasis target populations with potential to reduce anti-*Wolbachia* curative therapy times to between one and two weeks.

## Introduction

Lymphatic Filariasis (LF) and onchocerciasis are vector-borne diseases that endure as public health problems despite the efforts of sustained elimination programmes^1–3^. LF remains wide-spread throughout the tropics, affecting 120 million individuals with 1.1 billion at risk of infection^4^. It is symptomatically characterised by lymphoedema, hydrocele and elephantiasis and is the second leading cause of global disability^5^. Onchocerciasis is endemic in much of Sub-Saharan Africa, as well as more limited foci in Brazil, Venezuela and The Yemen with 37 million infected^6,7^. Onchocerciasis is the cause of skin disease and, in its most severe presentation, a sclerosing ocular keratitis (river blindness) which affects 0.8 million individuals and is the second most prevalent cause of infection-related preventable blindness^7–9^. LF and onchocerciasis are both targeted for elimination as public health problems^10,11^. The current strategy of elimination is mass drug administration (MDA) with anti-filarial drugs, which target the transmissive first-stage larvae (microfilariae; mf) produced by mating adult filariae^2,3,12^ For LF, the standard treatment is once single annual treatment with diethylcarbamazine (DEC) and albendazole (ABZ) in Asia, South America and Polynesia^13^. Due to contraindications of DEC in areas of potential overlapping onchocerciasis distribution in Africa, DEC is contraindicated (due to risk of severe ocular adverse reactions)^13,14^ and ivermectin (IVM) is substituted in combination with ABZ^15–18^. IVM is also used as a monotherapy in once- or twice-annual MDA for the elimination of onchocerciasis^19,20^. Because standard anti-filarial drugs given in these dosages and/or combinations have little macrofilaricidal activity, they have to be administered with high population coverage and repetitively over many years in order to break the transmission cycle of the long-lived, reproductively active adult filarial infections. This is predicted as at least five annual rounds for LF and fifteen annual rounds for onchocerciasis^2,3,16,17,20^, with the former a minimum timeframe to initiate transmission assessment surveys.

MDA has undoubtedly both reduced the burden of onchocerciasis disease and achieved nationwide elimination outcomes for LF^21–28^ in certain country settings. In other countries, however, failures of the approach are manifest. ‘Hot-spot’ residual foci of infections persist despite sustained coverage due to emerging resistance or other factors^29–32^, in certain regions, poor adherence to treatment is recorded, in part due to incidence of severe neurological adverse reactions to *Loa loa* co-infections^33,34^ in hard-to-reach areas, inadequate coverage is apparent and certain country elimination programmes are yet to commence^4,7^ In these scenarios, if LF and onchocerciasis elimination targets within ambitious 2030 United Nations Sustainable Development Goal (SDG) timeframes are to be achieved, there is an urgent need to implement alternative strategies. Affordable registered drugs, which have evidence of curative activity against filariae, and could be re-purposed to cure target populations within a test-and-treat delivery, are considered the most expeditious solution toward achieving elimination targets where deployed to mop up residual foci during the elimination ‘end-game’.

The antibiotic, doxycycline, has been shown to indirectly exert significant macrofilaricidal activity by targeting the filarial endosymbiotic bacteria *Wolbachia*^34,35^. Through a series of clinical trials in LF^36–41^ and onchocerciasis patients^42–45^, sustained, >90% depletion of *Wolbachia* from filarial tissues consequently mediates inhibition of embryogenesis, infertility, clearance of mf from the blood or skin and ultimately death of the adult filariae in 18–24 months^35,46^. Effects of depleting *Wolbachia* within migratory mf have also been demonstrated to hinder development to the infectious larval stage in intermediate vectors^47^. Sustained amicrofilaraemia/amicrofilaridermia following a single course of doxycycline^48,49^ leads to symptomatic relief and halts disease progression in onchocerciasis and interrupts transmission in both LF and onchocerciasis. Dose regimen reductions during clinical studies and subsequent modelling of curative trial outcomes for onchocerciasis^50^ have defined that doxycycline achieves significant macrofilaricidal activity via >90% *Wolbachia* depletion in both LF^48,49,51^ and onchocerciasis^42,44,52^ after 4 to 6 weeks of daily administration, dependent on dose (100 or 200 mg) and the target organism (*Wuchereria, Brugia* or *Onchocerca*).

However, two major impediments remain for the implementation of an anti-*Wolbachia* therapeutic approach based on doxycycline. The long treatment duration of 4 weeks minimum imposes a logistical challenge and risk poor adherence, whereby shorter durations of doxycycline treatment, although achieving significant *Wolbachia* reductions and impacting on embryogenesis, fail to achieve significant curative outcomes^53,54^. Secondly, doxycycline is contraindicated in significant proportions of the population such as in pregnancy and children of 8 years of age or younger^55^.

In this work, a strategy for achieving the equivalent efficacy of long-course doxycycline treatment by using a high dose of rifampicin for 1–2 weeks is presented. For this, preclinical *in vivo* mouse models of *Brugia malayi* or *Onchocerca ochengi* adult worm infections^56^, are used to compare the activity of different orally administered dosages to establish a PK-PD relationship of rifampicin that is translatable to humans. Using these empirical rifampicin experiments and PK-PD analysis we provide preclinical evidence to justify phase II trials re-purposing high dose rifampicin to deliver curative outcomes in periods of between 1 to 2 weeks.

## Results

### Intrinsic anti-*Wolbachia* potency of rifampicin is superior to doxycycline

Table 1 shows in its first row, the *in-vitro* IC_50_ levels obtained for doxycycline and rifampicin in the *Wolbachia* infected C6/36 cell (C6/36 *w*AlbB) assay^57^. Rifampicin exhibited an EC_50_ of 1.3 nM, which was approximately 16.2 fold more potent than that of doxycycline (EC_50_ = 22 nM), against *Wolbachia*. Both drugs resulted in killing ~90% of *Wolbachia* in comparison to vehicle control cell cultures at the end of the 7 day experiment.

**Table 1:** *In-vitro* and *in-vivo* IC50 values of doxycycline and rifampicin calculated based on the individual potency of each drug in the dose escalation studies in animals using PK-PD modelling.

Drug	Doxycycline	Rifampicin	Ratio (doxycycline/rifampicin)
*in-vitro* IC_50_ (mg/mL)	22	1.3	16.9
*in-vivo* IC_50_ (µg/mL)	1059	65	16.3

### Pharmacokinetics of rifampicin are superior to doxycycline

**Figure 1:**
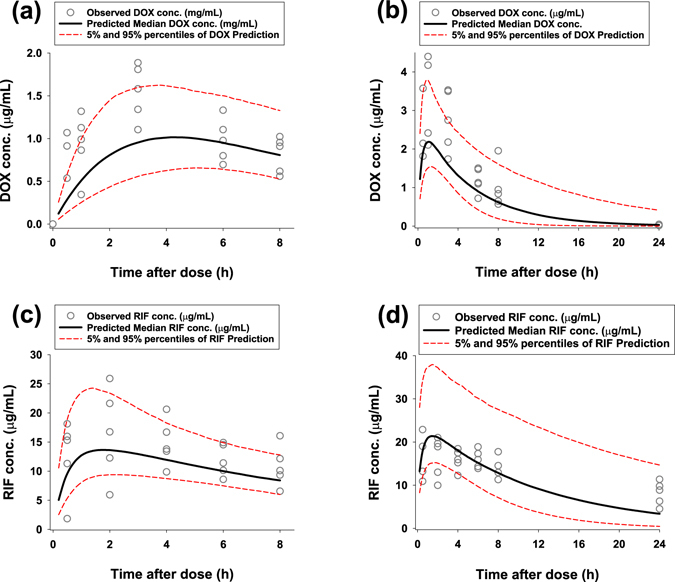
Pharmacokinetic profiles. Systemic exposure to doxycycline (DOX) and rifampicin (RIF) in BALB/c SCID mice are shown as follows. (**a**) PK profile of DOX after single 25 mg/kg dose. (**b**) PK profile of DOX at day 7 after 7 bi-daily 25 mg/kg doses. (**c**) PK profile of RIF after single 25 mg/kg dose. (**d**) PK profile of RIF at day 7 after 7 bi-daily 25 mg/kg doses.

Figure 1 shows the pharmacokinetic profiles measured in SCID mice at days 1 and 7 for doxycycline or rifampicin at a dose of 25 mg/kg *bid*. Table 2 summarises the pharmacokinetic parameters of each drug. Rifampicin (CL/F = 0.11 L/hr/kg) exhibited ~10 fold higher exposure at the same dose than doxycycline (CL/F = 1.2 L/h/kg). Clearance values at day 1 and day 7 were not statistically different (Mann-Whitney test, n = 5 mice, *P* > 0.05) and as shown in Table 2 all pharmacokinetics parameters of both drugs did not significantly change after chronic exposure in comparison to single dose administration.

**Table 2:** Pharmacokinetic parameters of doxycycline and rifampicin and after single dose and chronic dose administration.

Drug	Doxycycline		Rifampicin	
**Duration**	Single	7 day	Single	7 day
**Dose** (mg/kg)	25 mg/kg	25 mg/kg *bid*	25 mg/kg	25 mg/kg *bid*
**CL/F** (L/hr/kg)	1.9	1.4	0.11	0.128
**V/F** (L/kg)	7.0	8.2	1.45	1.551
**AUC** (mg.h/L) (0–8 h)	12.2	15.6	90.9	81.3

### Anti-*Wolbachia* activity of rifampicin is superior to doxycycline in adult filarial infection models

**Figure 2:**
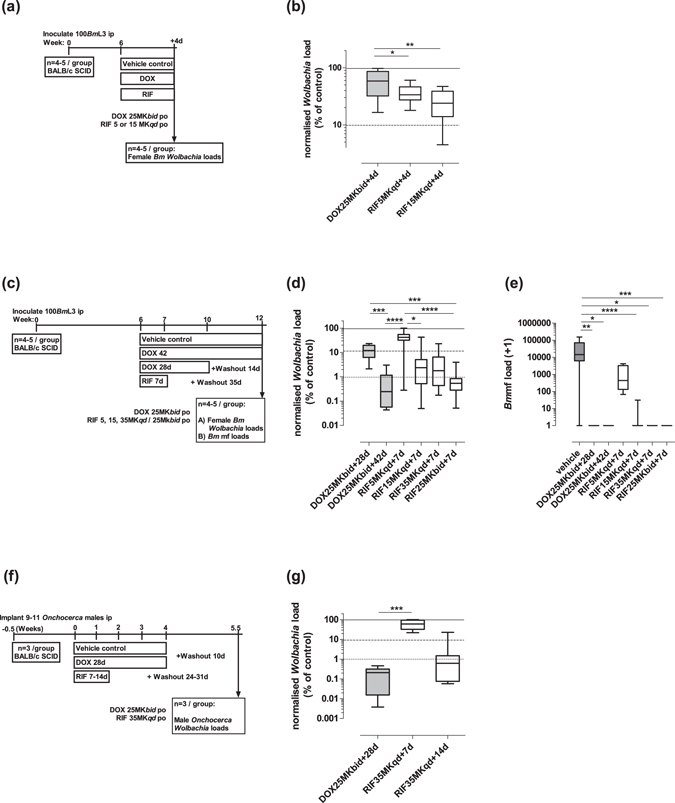
Pharmacological Outputs: Anti-filarial, anti-*Wolbachia* pharmacological effects of doxycycline (DOX) and rifampicin (RIF) *in vivo*. immediate pharmacological effects of DOX and RIF on *Brugia malayi* female adult *Wolbachia* loads (**a**,**b**) or post-washout (**c**,**d**). Effects on *B. malayi* microfilariae (*Bm*mf) production (**e**). Effects on *Onchocerca ochengi* male adult *Wolbachia* loads post-washout (**f**,**g**). Box and whiskers represent min-max (n = 10–29; *B. malayi Wolbachia*, n = 4–25; *Bm*mf, n = 5–17 *O. ochengi Wolbachia*). Solid line = control median *Wolbachia* level, long dashed line = 90%, short dashed line = 99% depletion level. Significance indicated *P < 0.05, **P < 0.01, ***P < 0.001, ****P < 0.0001.

We compared the anti-*Wolbachia* activity of rifampicin (5 mg/kg *qd*, 15 mg/kg *qd*, 35 mg/*kg qd* or 25 mg/kg *bid*) with doxycycline (25 mg/kg *bid*) in *B. malayi* and *O. ochengi* SCID mouse models (Fig. 2). The murine doxycycline dose had been previously defined as bioequivalent to clinical dosing with 100 mg/day^58^. *Wolbachia* rate-of-kill was examined by four day dosing of mice infected with adult-stage *B. malayi* followed by termination +24 h after last dose and retrieval of female adults for assessment of *Wolbachia* loads (Fig. 2a). Changes in *Wolbachia* post-dosing were calculated as a % reduction compared with the median level in the vehicle control group. Bioequivalent doxycycline mediated a 41.3% reduction in *Wolbachia* after +4 day exposures. In comparison, low dose rifampicin (5 mg/kg *qd*) mediated a significantly higher 66.3% reduction over the same time period (1 way ANOVA F = 7.561, *P* = 0.002, Dunnett’s multiple comparisons test *P* < 0.05 *vs* DOX; Fig. 2b) whilst high dose rifampicin (15 mg/kg *qd*) mediated a 76.0% median reduction (*P* < 0.01 *vs* DOX; Fig. 2b).

Elevations in oral rifampicin dose were undertaken to evaluate whether >90% threshold level *Wolbachia* depletions in female filarial tissues could be achieved in a time frame of seven days dosing. Six to seven week-old *B. malayi* timed infections were targeted with an additional washout period of five weeks to allow for continued depletion or recrudescence of the *Wolbachia* population to be evident, as previously reported^59,60^) (Fig. 2c). Doxycycline, at human bioequivalent (100 mg/day) dose^58^, was administered for four or six weeks (the latter a clinical dose time frame proven to deliver >90% *Wolbachia* depletion in *B. malayi*^39^ to compare with the efficacy of increasing dosages of short-course rifampicin regimens. As expected for ‘slow-killing’ anti-*Wolbachia* macrofilaricides, there were no significant changes in *B. malayi* worm burdens in either doxycycline or rifampicin dosed mouse groups compared with vehicle controls (Table 3).

**Table 3:** Adult *B. malayi* or *O. ochengi* worm burden recoveries post-dosing.

Treatment Species	Vehicle *B. malayi*	DOX *B. malayi*		RIF *B. malayi*				Vehicle *O. ochengi*	DOX *O. ochengi*	RIF *O. ochengi*	
**Dose (mg/kg)**	—	25	25	5	15	35	25	—	25	35	35
**Doses/day**	—	bid	bid	qd	qd	qd	bid	—	bid	qd	qd
**Duration (d)**	28–42	28	42	7	7	7	7	28	28	7	14
**SCID mouse (n)**	25	5	4	4	12	4	9	6	3	3	3
**Total worm (n)**	248	27	67	73	34	78	100	19	13	5	7
**Min**	0	0	14	0	0	15	4	0	3	0	1
**Median**	9	6	24	23	2	20	7	3	4	0	1
**Max**	34	10	29	27	9	23	21	6	6	5	5
**Female** ***B. malayi*** **(n)**	159	17	49	54	22	51	50	—	—	—	—
**Min**	0	0	11	11	0	0	0	—	—	—	—
**Median**	6	4	15	12.5	1	16.5	5	—	—	—	—
**Max**	23	7	23	18	6	18	12	—	—	—	—
***Wolbachia*** **sample (n)**	53	15	10	10	21	10	29	17	11	5	7

Doxycycline mediated an 88.1% median reduction in *Wolbachia* after four week dosing and 99.76% median reduction after six week dosing. In comparison, low dose rifampicin (5 mg/kg *qd* x 7d) was sub-optimal and statistically inferior to doxycycline, with a 58.3% median reduction (Kruskal-Wallis test *P* < 0.0001, Dunn’s multiple comparisons test, *P* < 0.0001 *vs* +42d DOX; Fig. 2d). Elevation of rifampicin dose to 15 mg/kg *qd*, 35 mg/kg *qd* or 25 mg/kg *bid* delivered 97.7%, 98.2% or 99.5% median *Wolbachia* reductions, respectively. These dose effects were statistically non-inferior to six-week doxycycline dosing. Rifampicin 25 mg/kg *bid* dosing for seven days displayed significantly superior anti-*Wolbachia* activity to four-week doxycycline (*P* < 0.0001) and 15 mg/kg *qd* or 25 mg/kg *bid* rifampicin were superior to 5 mg/kg rifampicin after seven day dosing (*P* < 0.05 and *P* < 0.0001, respectively; Fig. 2d). Examining the impact of anti-*Wolbachia* efficacy on mf release, four or six-week doxycycline mediated a complete absence of viable mf within the peritoneal infection site (5/5 or 4/4 mice mf negative *vs* 2/25 mf negative vehicle; Kruskal-Wallis test *P* < 0.0001, Dunn’s multiple comparisons test, *P* < 0.01 or *P* < 0.05 *vs* vehicle; Fig. 2f). Low-dose rifampicin (5 mg/kg *qd*) did not significantly affect the peritoneal *B. malayi* mf burden compared with vehicle control levels (0/4 mice mf negative; Fig. 2e). In comparison, seven day dosing with 15 mg/kg *qd* rifampicin mediated a near complete absence of viable mf from the peritoneum of infected mice (11/12 mice mf negative; *P* < 0.0001 *vs* vehicle; Fig. 2e). Seven-day doses of rifampicin exceeding 15 mg/kg *qd* mediated total absence of viable mf (35 mg/kg *qd*: 4/4 mice mf negative *P* < 0.05 *vs* vehicle, 25 mg/kg *bid*: 9/9 mice mf negative *P* < 0.001 *vs* vehicle; Fig. 2e).

Based on threshold effects of elevated dose rifampicin achieving >90% *Wolbachia* depletions from *Brugia* adult female tissues following seven day dosing, we tested efficacy against *Onchocerca* male worms in a novel SCID mouse model of onchocerciasis^56^ Rifampicin dosed at 35 mg/kg *qd* for seven or 14 days was compared with human bioequivalent doxycycline dosed for 28 days (a minimum clinical dose/duration determined to deplete >90% *Wolbachia* from adult *O. volvulus* tissues leading to macrofilaricidal activity^50^, (Fig. 2f). Male *Onchocerca* worm burdens were not affected by treatment compared with vehicle controls (Table 3). Bioequivalent doxycycline mediated 99.6% median reduction in *Wolbachia* from male *Onchocerca* tissues following 28 days dosing (Fig. 2g). Seven-day dosing of rifampicin did not mediate any depletion in *Wolbachia* following washout period (0% median reduction) and was thus statistically inferior to doxycycline control (Kruskal-Wallis test *P* = 0.0013, Dunn’s multiple comparisons test, *P* < 0.001 *vs* DOX; Fig. 2g). In comparison, 14 day dosing of rifampicin mediated 96.9% median reductions in *Onchocerca Wolbachia*, a level of efficacy that was statistically non-inferior to doxycycline dosed for 28 days (Fig. 2g).

### PK-PD modelling

The empirical PK and pharmacological anti-*Wolbachia* efficacy measurements of doxycycline and rifampicin were used to construct a PK-PD model (described in the methods section). An *in vivo* IC_50_ value was calculated based on the model for rifampicin (65.2 µg/ml), which was ~16-fold more potent than that calculated for doxycycline (1059 µg/ml; Table 1). IC_50_ values derived from the PK-PD model were 100,000 to 200,000 fold higher than those observed *in vitro*; however the ratio of calculated *in vivo* IC_50_ between rifampicin and doxycycline was aligned to the ratio empirically measured *in vitro* between the two drugs (~17-fold higher RIF *vs* DOX, Table 1).

### Mouse – Human Bridging analysis

**Figure 3:**
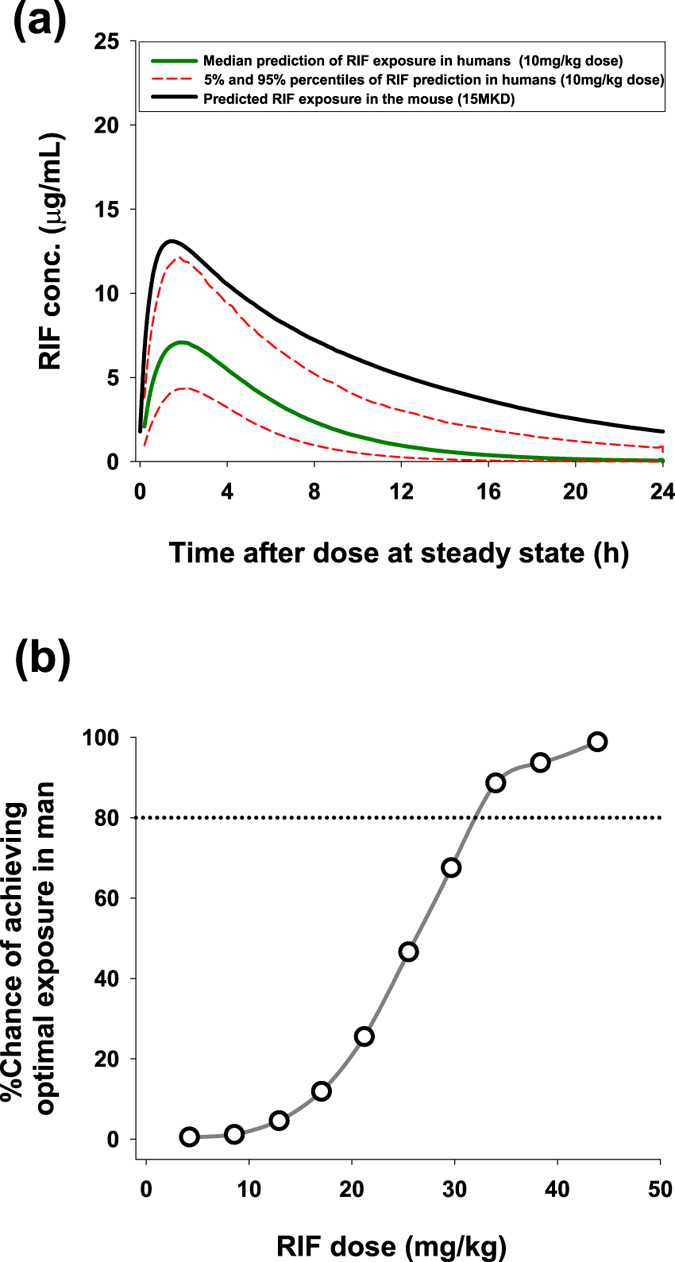
Human-Mouse Bridging. PK profile of rifampicin in mouse at 15MKD (Black solid line) in comparison to exposures expected in man based on 600 mg dosing in 70 kg individuals (**a**) (green solid line representing median prediction and dashed red lines representing 5% and 95% percentiles of the prediction). % Probability of achieving optimal rifampicin exposure in humans at a dosing range of 4 mg/kg–45 mg/kg (**b**). (optimal exposure is defined as the median total 24 hour steady state AUC in mice receiving daily 15 mg/kg dose).

To compare rifampicin exposures observed in the SCID mouse model in comparison to those achieved in standard human clinical dose (600 mg), the overall exposure of rifampicin has been estimated in humans based on literature data (Fig. 3a). From this we determined the exposure in an average population of 70 kg humans after receiving 600 mg dose of rifampicin is significantly lower than the optimal exposure in mice that results in >90% *Wolbachia* elimination in 7 days or less (this exposure is defined as the 24 hour steady state AUC of the 15 mg/kg rifampicin dose in mice which is 141.5 mg/hr/L). The human dose that would achieve this optimal mouse exposure was then determined via multiple Monte-Carlo simulations (3000 subjects per dose) at different doses from 5 mg/kg up to 45 mg/kg where the percentage of humans predicted to achieve the desired AUC at each dose has been recorded (Fig. 3b). The PK predictions of higher doses of Rifampicin agree with published results where the predicted C_max_ and AUC were equivalent to observed PK in humans at elevated doses in clinical studies^61^. This would indicate that our dosing suggestion will achieve the desired exposure based on clinical PK data.

From those simulations, it is predicted that a minimum dose of 30–40 mg/kg is needed to achieve ‘curative’ exposure levels in human filariasis patients which also agrees with high dose RIF clinical studies^61^ that have shown a dose of 30 mg/kg or higher to achieve a steady-state AUC of 141.5 mg/hr/L in the vast majority of patients

## Discussion

Targeting *Wolbachia* with antibiotics to deliver curative outcomes for filariasis is a promising drug development strategy. The pharmacopeia of registered antibiotics present ‘low hanging fruit’ with the potential for rapid re-purposing^62,63^. Further, the ‘slow kill’ effect post-*Wolbachia* depletion, manifest first on circulating or skin mf and then adult worms, avoids the problems of inflammatory adverse reactions which are associated with fast-killing direct filaricidal agents^3,33,64–66^. Indeed, this promotes anti-*Wolbachia* therapies as a safe drug development strategy for to administer to *L. loa* co-endemic target populations^67^ who are at risk of severe neurological adverse reactions to rapid acting filaricides and thus provides a potential solution to problem areas of Central Africa toward an end game of onchocerciasis elimination^68^. Clinical trials with doxycycline have shown that depletion of *Wolbachia* from filarial nematodes can deliver significant macrofilaricidal activity when administered for 4 or more weeks^38,40–42,51^. However, the long period of administration and contraindications in children and pregnant women present logistical challenges to widespread scale-up in resource poor settings of Sub-Saharan Africa. Whilst we have recently identified minocycline as a superior anti-*Wolbachia* agent to doxycycline in the *B. malayi* SCID mouse model^58^ as part of the same tetracycline class, the same contraindications would apply. The ‘holy grail’ for an anti-*Wolbachia* macrofilaricide is thus demonstrable activity in administration times of a minimum of 2 weeks (ideally 1 week) and a safety profile for administration in loiasis co-infections, in children and in pregnant women^34,63^.

We use here a validated screening system of *in vitro* cell assays and *in vivo* preclinical mouse models of filariasis^56,57^ to test the efficacy of dose elevations of rifampicin and have identified a dose-dependent activity which has the potential to achieve *Wolbachia* depletions predictive of cure when administered for 1 week against the human lymphatic filaria *B. malayi* or 2 weeks against *O. ochengi*, the closest phylogenetic relative of *O. volvulus*.

We determine that rifampicin is >15 fold more potent than doxycycline *in vivo*. Rifampicin exhibited significantly higher exposure per mg dose when compared to doxycycline. Also PK-PD modelling showed that rifampicin is more potent in reducing *Wolbachia in vivo* when exposure levels are matched to doxycycline. This superiority likely reflects the inherently higher (>16 fold) intrinsic potency of rifampicin against *Wolbachia* that is observed *in vitro* compared with doxycycline.

Corroborating rifampicin superiority versus doxycycline as an anti-*Wolbachia* agent, it has previously been reported, in mice experimentally infected with the rodent filaria, *Litomosoides sigmodontis*, that 25 mg/kg *bid* dosages of rifampicin result in a more profound decrease in *Wolbachia* levels in adult worms when compared to a similar dose of doxycycline^48^. Also, in a pilot study using the related, registered rifamycin, rifapentine, >90% depletion of *Wolbachia* was measured in *O. ochengi* males implanted into SCID mice, following two weeks 25 mg/kg *bid* dosing^56^. However, clinical studies in humans have shown that rifampicin administered for two or four weeks has inferior activity to six-week doxycycline in onchocerciasis^69,70^ and neither is combining rifampicin and doxycycline for two weeks sufficient to achieve significant macrofilaricidal activity^54^.

Our PK-PD model offers a rational explanation for the discrepancy in the activity of rifampicin in mice and humans, where doxycycline activity is similar across mouse model and human clinical studies, yet rifampicin seems to be dramatically more active in the mouse than in humans. We identify effective dosages in the mouse exceed the exposure of rifampicin achieved in humans when the drug is administered at ‘standard dose’ (~10 mg/kg *qd* or 600 mg). Indeed, exploring dose ranges more in line with standard human dose exposures, (5 mg/kg *qd*) rifampicin in the mouse is insufficient to achieve >90% *Wolbachia* depletion in 1 week against *B. malayi.*

The currently established 600 mg dose of rifampicin has not been predicated on PK-PD analysis but rather has been chosen based on drug cost, due to rifampicin semi-synthetic production in the 1970 s when the dosage was established^71^. However, recently the cost of rifampicin production has dramatically decreased and dose escalation studies have been initiated for TB patients. Higher doses of rifampicin (up to 35 mg/kg) have recently been proven to be safe in a clinical study^61^. The same study undertook human PK analysis and has shown that rifampicin exposure increases as the dose is increased. From this data we predict that the target exposure level required to deplete >90% *Wolbachia* from target filarial species in 1–2 weeks to ultimately deliver short-course curative efficacy against filariasis is achievable in humans when a dose of 30–35 mg/kg is administered. Importantly, clinical data supports that for doses of up to 35 mg/kg, given for periods over 2 weeks, no serious side effects are observed and that grade 1 and grade 2 side effects are randomly distributed across low and higher doses indicating no increased risk of side effects with elevated doses of rifampicin. A recent additional study where a dose of 35 mg/kg of RIF was administered to TB patients has also reported similar conclusions in relation to safety^72^.

Because elevated rifampicin dosages for filariasis cure are predicted in a 1–2 week dose exposure time frame, this is unlikely to result in resistant strains of *M tuberculosis* (TB), as there is no evidence for rifampicin resistance occurrence when administered for short periods of time^73,74^.

Rifampicin has a number of drug-drug interactions of clinical significance that have been previously reported^75^. Those interactions can result in the reduction of exposure of other drugs that are taken concomitantly with Rifampicin, and doses might need to be altered, especially for patients who are on HIV medication.

## Conclusions

Using PK-PD modelling of drug activity in preclinical filarial mouse models, we show that a clinically safe dose of rifampicin can be administered for 1–2 week short courses to elicit >90% reductions in *Wolbachia*, predictive of the macrofilaricidal activity observed when *Wolbachia* is targeted by doxycycline, administered for 4–6 weeks. This treatment regimen would be compatible for use in children and during pregnancy and, because of the shortened duration of administration required, would be more readily deliverable by health care systems in resource-poor community settings. A 35 mg/kg dose has been proven as safe over a period of 2 weeks, and it has previously been shown that such short durations of treatment will not produce resistant strains of *M. tuberculosis*. We recommend that both a 35 mg/kg 1 and 2 week dose of rifampicin be urgently examined in lymphatic filariasis and onchocerciasis randomised controlled clinical trials to test whether such short-course treatment regimens are indeed sufficient to elicit equivalent curative activities to long course doxycycline.

## Methods

### Mice

Six to ten week old male BALB/c SCID (Harlan Laboratories, UK) or male CB.17 BALB/c SCID mice (Charles River, UK) were used for experiments. Mice were kept at the Biomedical Services Unit (BSU) at the University of Liverpool, UK, in specific pathogen-free (SPF) conditions or at The Research Foundation for Tropical Medicine and The Environment, Buea, Cameroon, in individually-ventilated caging. All experiments on animals were approved by The Animal Welfare and Ethical Review Board, Liverpool School of Tropical Medicine, UK, or the University of Buea Animal Ethics Review Board, Cameroon. Studies were conducted in accordance with Home Office legislation (UK) and matching welfare standards were applied in Cameroon.

### *In vitro* potency studies

The anti-*Wolbachia* potency of doxycycline and rifampicin for use in the PK-PD model was determined *in vitro*, utilising the routine A·WOL screening assay as described previously^57^. In brief the mosquito (*Aedes albopictus*) derived cell line C6/36 (ATCC number CRL-1660), stably infected with *Wolbachia pipientis* (*w*AlbB) (C6/36 *w*AlbB) was incubated with the relevant drugs in a concentration range in order to determine a dose response. The drugs were incubated for 7 days with 2,000 cells per well on a 384 well plate (CellCarrier-384 Ultra, PerkinElmer) in Leibovitz media (Life Technologies™) supplemented with 20% foetal bovine serum (FBS, Fisher Scientific), 2% tryptose phosphate broth (Sigma-Aldrich) and 1% non-essential amino acids (Sigma-Aldrich). The end-point read out utilised DNA staining of both the host cell nuclei and intracellular *Wolbachia* (SYTO® 11) combined with a high content imaging system (Operetta®, PerkinElmer) and analysed using the associated Harmony® software through a cytoplasm texture analysis.

### Brugia malayi experimental infections

The *Brugia malayi* lifecycle was maintained through mosquitoes and susceptible *Meriones* gerbils at LSTM. To generate infective *B. malayi* larvae for infections, female adult *Aedes aegypti* mosquitoes were fed with microfilariae collected from infected gerbils by catheterisation, as described previously^76^, that were mixed with human blood. Mosquitoes were fed through an artificial membrane feeder (Hemotek®). Blood-fed mosquitoes were reared for 14 days to allow for development to the L3 stage. L3 were then collected from infected mosquitoes by crushing and concentrated using a Baermann’s apparatus and RPMI medium. 100 motile L3 were collected and then injected into mice via the intra-peritoneal route. Efficiency of inoculations was confirmed by needle washout.

### *Onchocerca ochengi* surgical implantations

Viable male *Onchocerca ochengi* were aseptically isolated from naturally parasitized cattle as described previously^56^. Between 10–11 male *Onchocerca* were surgically implanted into the peritoneal cavity of CB.17(BALB/c) SCID mice under anaesthesia as described previously^56^.

### Drug treatments

Six weeks after *B. malayi* experimental infection or 3 days post *O. ochengi* surgical implantation, SCID mice received 100 µL compound via oral gavage at variable doses and for variable treatment lengths as described and indicated in further detail in the main text. The drug doses and routes used were: Doxycycline (25 mg/kg *qd* or *bid* po and rifampicin (1.25 mg/kg–25 mg/kg *qd* or *bid* po). Doxycycline was dissolved in water, while rifampicin was dissolved in 55% polyethylene glycol 300; 25% propylene glycol; 20% water. All drugs and vehicle reagents were purchased from Sigma Aldrich.

### Parasitological readouts

Seven or twelve weeks after *B. malayi* experimental infection or 38 days after *O. ochengi* surgical implantation, mice were necropsied. Adult filariae and released *B. malayi* mf were recovered by a combination of peritoneal washings using wash media (RPMI containing P/S) and subsequent dissection of abdominal cavities. To quantify *B. malayi* mf, samples were centrifuged at 1200 rpm for 5 min. Excess supernatants were removed and the remaining volume quantified. Three 20 µL volumes were then enumerated for mf by microscopy, scoring as motile or stretched immotile. Adult filariae were observed for motility, washed in cold PBS and parasite stages enumerated. A minimum of ten *B. malayi* females and five *O. ochengi* males per treatment group of 3–5 mice were collected into individual Eppendorf tubes to be processed for qPCR.

### DNA extraction and PCR quantification of Wolbachia

DNA was extracted from worm samples using a DNeasy Blood and Tissue Kit (Qiagen) according to manufacturer’s instructions. Levels of *Bm Wolbachia* surface protein (*wsp*) gene copy numbers were quantified using qPCR as previously described^77^. Levels of *Onchocerca Wolbachia wsp* and filarial *gst* gene copy numbers were similarly quantified as previously described^56^.

### PK Studies

To establish the pharmacokinetic profiles of rifampicin and doxycycline, rich pharmacokinetic studies were performed in uninfected male SCID mice (weight 24–28 g). Doxycycline (25 mg/kg *bid*) or rifampicin (5 mg/kg *qd* or 25 mg/kg *bid*) were administered orally for up to seven days. For all PK studies, a total of 11 samples were collected at discrete time points between 0 and 24 hour post dose at days 1 and 7). Serial blood samples were collected at days 1 and 7 of dosing via the tail vein where a microincision was performed and 20 µL of blood collected using a pipette with a pre-heparinised tip. Blood samples were directly lysed with 40 µL of ice cold ddH_2_O and then frozen at −80 °C until time of bioanalysis.

### Bioanalysis

Rifampicin concentrations were determined by LCMS (liquid chromatography mass spectrometry) using an appropriate internal standard and were validated to internationally recognised acceptance criteria [FDA-guidelines]. Chromatographic separation was achieved using a gradient programme. The UHPLC (ultrahigh pressure liquid chromatography) system was interfaced with a triple-quadruple TSQ Quantum Access mass spectrometer (Thermo Scientific, Hemel Hempstead, UK) with a heated-electrospray ionization (H-ESI) source. An E2M28 rotary vacuum pump (Edwards High Vacuum International, West Sussex, UK), an NM30LA nitrogen generator (Peak Scientific, Renfrewshire, UK) and 99% pure argon gas (10 L, BIP10, Air Products, Liverpool, UK) were used. Blood samples (20 μl) containing rifampicin were extracted with a mixture of acetonitrile and methanol (80:20 v/v). The subsequent mix was then filtered through 96 well filter plate (Millipore, UK). The resultant filtrate was then transferred to a 96 deep well plate containing 40 µl of (10 mg/ml) ascorbic acid, 10 µl of this resultant mixture was then injected into the LC-MS/MS. The assay was linear in the range 10–10,000 ng/ml for rifampicin.

### PK-PD Modelling

A PK-PD model as described in equations 1 and 2 has been developed and used to fit parasitological read-outs from experiments in infected mice. The pharmacological output for all PK/PD analyses was *Wolbachia* counts as estimated in qPCR experiments in the quantitation of *wsp* DNA levels. Pmetrics® ^78^ was used in modelling PK/PD data in all experiments. The following differential equations were used:

For the PK component:

1 $${C}_{p}=\,\frac{F\cdot DOSE\cdot {k}_{a}}{V\cdot ({k}_{a}-{k}_{e})}\cdot [{e}^{-{k}_{el}\cdot t}-{e}^{-{k}_{a}\cdot t}]$$

Where *C*_*p*_ is the drug concentration in blood at any given time, F is the fraction available for oral absorption, k_a_ is the rate of absorption from gut to blood, V is the volume of distribution and k_el_ is the rate of elimination from the systemic circulation.

For the PD component, a relationship was built to describe the effect of the drug upon *Wolbachia* levels using a dynamic *E*_*max*_ model:

2 $$Wolbachia\,count\,({X}_{1})=\,{X}_{1}\cdot ({k}_{growth}-\,\frac{{k}_{kill}\,\cdot \,{C}_{p}}{I{C}_{50}\cdot {C}_{p}})$$

*X*_*1*_ is the observed *Wolbachia* levels as estimated by qPCR, k_growth_ is the rate of bacterial growth which was set at 0 due to the absence of any evidence of bacterial growth beyond the time of treatment and no evidence of recrudescence within the time frame of the experiment. K_kill_ is the maximum possible rate of kill of bacteria and has been fixed to the *in vitro* kill rate that could be achieved at the maximum concentration of drug. IC_50_ is the blood concentration required to achieve 50% of the maximal kill rate of *Wolbachia* in the adult worm in the mouse.

### Statistics

PK parameters between doxycycline and rifampicin were compared by Mann-Whitney test. Percentage *Wolbachia* reductions in filariae following *in vivo* treatments were calculated from median vehicle control levels derived from the same experimental infection and screen. Where repeat experiment data (*Wolbachia* loads normalised to control, worm enumerations or viable mf enumerations per mouse) were available, data was pooled. Grouped continuous variables were tested for normal distribution by D’Agostino & Pearson omnibus normality tests. Continuous variables failing normal distribution tests were Log^10^ transformed and re-tested. Continuous variables satisfying the assumptions of normal distribution were examined by 1 way ANOVA with Dunnett’s multiple tests post-hoc. Variables not satisfying the assumption of normality were compared by Kruskal-Wallis Test with Dunn’s Multiple Tests, post-hoc. Significance levels are indicated *P* < 0.05*, *P* < 0.01** *P* < 0.001*** *P < *0.0001****. All statistics were undertaken using GraphPad Prism v6 software.
