# Author Correction: Short-Course, High-Dose Rifampicin Achieves *Wolbachia* Depletion Predictive of Curative Outcomes in Preclinical Models of Lymphatic Filariasis and Onchocerciasis

**DOI:** 10.1038/s41598-018-19723-1

**Published:** 2018-01-18

**Authors:** Ghaith Aljayyoussi, Hayley E. Tyrer, Louise Ford, Hanna Sjoberg, Nicolas Pionnier, David Waterhouse, Jill Davies, Joanne Gamble, Haelly Metuge, Darren A. N. Cook, Andrew Steven, Raman Sharma, Ana F. Guimaraes, Rachel H. Clare, Andrew Cassidy, Kelly L. Johnston, Laura Myhill, Laura Hayward, Samuel Wanji, Joseph D. Turner, Mark J. Taylor, Stephen A. Ward

**Affiliations:** 1 Department of Parasitology, Liverpool School of Tropical Medicine, Pembroke Place, Liverpool L3 5QA UK; 2 Research Foundation in Tropical Medicine and the Environment, Buea, Cameroon; 3 Department of Microbiology and Parasitology, University of Buea, Buea, Cameroon

Correction to: *Scientific Reports* 10.1038/s41598-017-00322-5, published online 16 March 2017

The original version of this Article contained a typographical error in the spelling of the author Haelly Metuge, which was incorrectly given as Haelly Metugene.

This has now been corrected in the PDF and HTML versions of the Article.

